# What is the best long-term treatment modality for immature permanent teeth with pulp necrosis and apical periodontitis?

**DOI:** 10.1007/s40368-020-00575-1

**Published:** 2021-01-08

**Authors:** A. Wikström, M. Brundin, M. F. Lopes, M. El Sayed, G. Tsilingaridis

**Affiliations:** 1 Division of Orthodontics and Paediatric Dentistry, Department of Dental Medicine, Karolinska Institutet, Huddinge, Sweden; 2 Public Dental Health Services, Department of Endodontics, Eastmaninstitutet, Stockholm, Sweden; 3 Centre of Paediatric Oral Health, Stockholm, Sweden; 4 Department of Paediatric Dentistry, Public Dental Health Services, Eastmaninstitutet, Stockholm, Sweden; 5 Department of Endodontics, Umeå University, Umeå, Sweden

**Keywords:** Open apices, Pulp necrosis, Pulp regeneration, Regenerative endodontics, Immature teeth, Children

## Abstract

**Purpose:**

To evaluate and assess the current knowledge about apexification and regenerative techniques as a meaningful treatment modality and to map the scientific evidence for the efficacy of both methods for the management of traumatised immature teeth with pulp necrosis and apical periodontitis.

**Methods:**

This systematic review searched five databases: PubMed, Web of Science, Cochrane Library, Ovid (Medline), and Embase. Published articles written in English were considered for inclusion. The following keywords were used: Regenerative endodontic treatment OR regenerat* OR revital* OR endodontic regeneration OR regenerative endodontics OR pulp revascularization OR revasculari* OR ‘traumatized immature teeth’. Only peer-reviewed studies with a study size of at least 20 cases followed up for 24 months were included. Eligibility assessment was performed independently in a blinded manner by three reviewers and disagreements were resolved by consensus. Subgroup analyses were performed on three clinical outcomes: survival, success, and continued root development.

**Results:**

Seven full texts out of 1359 citations were included and conventional content analysis was performed. Most of the identified citations were case reports and case series.

**Conclusions:**

In the present systematic review, the qualitative analysis revealed that both regenerative and apexification techniques had equal rates of success and survival and proved to be effective in the treatment of immature necrotic permanent teeth. Endodontic regenerative techniques appear to be superior to apexification techniques in terms of stimulation of root maturation, i.e. root wall thickening and root lengthening. Knowledge gaps were identified regarding the treatment and follow-up protocols for both techniques.

**Electronic supplementary material:**

The online version of this article (10.1007/s40368-020-00575-1) contains supplementary material, which is available to authorized users.

## Introduction

Traumatic dental injuries to permanent dentition are a global health issue and are the most common aetiological factor for pulp necrosis. Trauma is estimated to affect one billion people worldwide (Petti et al. 2018), and one-third of these patients have injuries to their immature teeth that might cause pulp necrosis (Hecova et al. 2010). Pulp necrosis arrests root formation where the root remains short and the dentin walls thin, increasing the risk for cervical fractures (Tsilingaridis et al. 2012). The combined effect of these factors makes it challenging to perform an adequate antibacterial endodontic treatment (Kakoli et al. 2009; Orstavik and Haapasalo 1990).

In recent years, much effort has been made to find alternatives to conventional endodontic treatment since it does not lead to further root formation, leaving the tooth susceptible to root fracture and possibly to the loss of the entire tooth. Furthermore, the most serious sequelae of early tooth extraction are the loss of volume of the alveolar process, which leads to difficulties in future prosthetic treatment planning. The problem is that the open apices of traumatised immature teeth make it challenging to perform an adequate root filling without intra-operative complications, such as extrusion of root-filling material into the periapical tissue, s.c. overfilling (Trope 2010).

### Current available treatment modalities for immature necrotic permanent teeth

Different techniques have been adopted to prevent overfilling and to stimulate the closure of the apical area—s.c. apexification techniques. A widely accepted endodontic approach in the treatment of immature teeth with pulp necrosis and apical periodontitis is the apexification technique. This technique consists of multiple and long-term applications of calcium hydroxide (CaOH_2_) (Petti et al. 2018). Many studies show continued apical formation by hard tissue deposition where the teeth are asymptomatic with radiographical signs of closure of the apical foramen (Hecova et al. 2010; Tsilingaridis et al. 2012). The main advantage of this technique is the excellent disinfecting effect of the root canal due to the high pH of CaOH_2_ (Kakoli et al. 2009; Orstavik and Haapasalo 1990). On the other hand, this technique often requires 9–24 months of treatment (Sheehy and Roberts 1997). In addition, CaOH_2_ may weaken the tooth structure, which often results in cervical root fractures (Andreasen et al. 2002). Understandingly, many studies discuss the consequences of premature tooth loss and highlight the urgent need for alternative treatment strategies for this patient group (Orstavik and Haapasalo 1990; Trope 2010; Sheehy and Roberts 1997; Andreasen et al. 2002; Dawood et al. 2017).

As an alternative to apexification with CaOH_2_, another technique has been introduced that stimulates apical hard tissue formation to aid obturation of teeth with open apices—the s.c. MTA-apical plug technique (mineral trioxide aggregate, MTA). Many studies using this technique report good results in periapical healing and signs of closure of the apical foramen (Mozynska et al. 2017; Chueh and Huang 2006). The MTA technique has the advantage of shorter treatment time as well as being biocompatible, which improves the interaction with the periapical tissue such as induction of cell proliferation and differentiation (Murray et al. 2007; Hargreaves et al. 2008). However, MTA does have several drawbacks. The MTA’s interaction with collagen causes tooth discoloration (Dawood et al. 2017), an undesirable consequence that may be the result of bismuth oxide and iron contamination of the blood (Mozynska et al. 2017). In addition, little is known about how the material influences the fracture resistance in immature traumatised teeth (Hecova et al. 2010; Tsilingaridis et al. 2012).

Neither calcium hydroxide nor MTA-apical plug apexification improves root formation and thickening of the dentin walls and therefore does not lead to the strengthening of the immature tooth and better long-term survival (Trope 2010; Sheehy and Roberts 1997). Hence, it is of high priority to replace conventional endodontic procedures with a new biological approach that will stimulate regeneration of the periapical tissue and continued root formation (Chueh and Huang 2006; Murray et al. 2007; Hargreaves et al. 2008; Lovelace et al. 2011). The ultimate goal of the treatment of traumatised immature teeth is to prolong the life span of the traumatised tooth and to postpone or prevent the need for more extensive prosthetic alternatives (Op Heij et al. 2003).

Recently, regenerative endodontic treatments (RET) have gained much attention as biologically based treatment alternatives to the techniques described above. These regenerative endodontics, which are gradually becoming part of the endodontic treatment spectrum, replace necrotic and damaged tissues with a healthy functioning pulp–dentin complex (Garcia-Godoy and Murray 2012). As regenerative endodontic treatment is a relatively new treatment modality, little is known about the processes about the type of intra-canal tissues formed during revascularisation and about the factors involved in healing and regeneration (Žižka and Šedý 2016).

### Significance of the review

Although the interest in regenerative endodontic treatment is increasing significantly, it is currently not the first choice for the management of immature permanent teeth with pulp necrosis as scientific evidence has not established the efficacy of the method (Rafter 2005; Cvek 1992; Trope 2010) and little is known about the long-term outcome. Therefore, it would be beneficial to evaluate and assess the current knowledge about this technique as a meaningful treatment option. Many studies have investigated the field of endodontic regeneration, but unfortunately most of these are based on small study sizes (*n* = 1–5) (Asgary et al. 2016; Zhujiang and Kim 2016; Farhad et al. 2016; Nagaveni et al. 2016; Nagaveni et al. 2015; Wang et al. 2015) and/or short follow-up (6–12 months) (Hargreaves et al. 2013; Gelman and Park 2012; Kim et al. 2012; Jadhav et al. 2012). Short follow-ups are a limitation because severe complications, such as ankylosis, can occur as a consequence of traumatic injuries to immature permanent teeth the first 2–3 years after an injury (Lauridsen et al. 2019). Therefore, the dental trauma itself influences the long-term prognosis rather than the type of treatment performed. Consequently, an internationally accepted approach is to postpone the completion of the endodontic treatment until the growth of the jaws is complete. This fact emphasises the need for intervention studies with sufficient study sizes and follow-up periods in order to find evidence for best practices.

To date, several review articles have been written on the subject of regenerative endodontic treatment (Antunes et al. 2016; Nicoloso et al. 2019; Nazzal and Duggal 2017; He et al. 2017). These reviews investigate and propose different important outcomes, such as quantitative measurements of tooth-root development, resolution of apical periodontitis, and surrogate outcome measurements of survival or success. To our knowledge, no systematic reviews have focused exclusively on long-term studies of RET (≥ 24 months) with larger study sizes (≥ 20 patients). For the reasons outlined above, this review critically evaluates whether regenerative endodontics can routinely be recommended in the treatment of traumatised immature teeth with pulp necrosis and infection assuming that the treatment modality will benefit the long-term survival of traumatised teeth and eventually the well-being of the patient.

## Aim and objectives of the review

This review investigates what aspects of the treatment of immature necrotic permanent teeth should inform best practices guidelines of the Centre of Paediatric Oral Health (Stockholm, Sweden). Specifically, this review maps and evaluates whether regenerative endodontic treatment in traumatised immature permanent incisors with pulp necrosis and apical periodontitis produces better long-term results than conventional endodontic treatment with apexification techniques with calcium hydroxide or MTA. In addition, this review identifies the critical issues that need guidance and determines the key outcomes and measurements of outcomes.

A focused question was developed in accordance with the Patient, Intervention, Comparison, and Outcome (PICO) method (SBU 2017):

(P) Participants/population: Immature necrotic permanent teeth.

(I) Intervention(s): Endodontic treatment performed on immature necrotic permanent teeth treated with RET techniques.

(C) Comparator(s)/control: Conventional endodontic treatment of immature necrotic permanent teeth with CaOH_2_ /MTA apical plug techniques.

(O) Outcome: Clinical and radiographic—symptoms, periapical healing, dentin wall thickening, apex closure, continued root development, and discoloration.

### Research questions

1. Which aspects in the treatment of immature necrotic permanent teeth influence the outcome of regenerative endodontic procedures and apexification techniques with calcium hydroxide or MTA apical plug techniques?
2. What measures have been used to assess the treatment outcomes of regenerative endodontic procedures and apexification techniques with calcium hydroxide or MTA apical plug techniques?
3. What is the current scientific evidence for regenerative endodontic treatment in traumatised immature permanent incisors with pulp necrosis and apical periodontitis?
4. What is the current scientific evidence for conventional endodontic treatment with apexification techniques with calcium hydroxide or MTA for the same patient group?

## Methods

### Review type

Prior to conducting the work with the current review, a scoping search was performed on the International prospective register of systematic reviews, PROSPERO in order to avoid duplication (registration number CRD42020204801). No matches were found on protocols related to the topic and the study was subsequently registered before completion (Stewart et al. 2012). The search process was carried out in two steps according to the guidelines for systematic reviews.

First, we mapped the subject (Grant and Booth 2009) as mapping provides a systematic description of the search, screening, and classification process, rendering a representative sample of published literature (Gough et al. 2012). The overall goal was to map, categorise, and synthesise the findings of the specific topic area.

Second, we appraised and synthesised the research evidence for endodontic treatment of traumatised immature teeth in order to insure that the literature study findings are an accurate representation of the studies it contains (Thomas 2005).

### Categorisation into subgroups of studies to be included

The eligible studies were categorised into two subgroups: studies of immature permanent teeth with pulp necrosis and open apex treated with regenerative endodontic treatment (RET studies) and studies of immature permanent teeth with pulp necrosis and open apex treated with CaOH_2_ or MTA apexification techniques (CaOH_2_ or MTA studies) (SBU 2017).

### Inclusion and exclusion criteria

#### Study design and setting

The following inclusion criteria were used: longitudinal studies, published in English, random controlled trials (RCTs), prospective and/or retrospective cohorts, and treatment of immature necrotic permanent teeth, follow-up of at least 20 teeth for 24 months (Table 1). In addition, as the rationale for RET is to stimulate continued root development, only studies on immature necrotic teeth in healthy individuals aged between 6 and 19 years were included. However, age restrictions in MTA were not set, as the rationale for the treatment is to obtain a mechanical ‘barrier’ for obturation rather than to stimulate continued root development.

**Table 1:** Inclusion and exclusion criteria

Literature inclusion
Peer-reviewed studies regarding immature permanent teeth with pulp necrosis and open apex including both clinical and radiographic outcomes. Follow-up of ≥ 20 teeth ≥ 24 months RET studies CH or MTA studies
Patients’ age in the RET studies: 6–19 years Patients’ age in the CH or MTA studies: Group 2: no restrictions
Studies published in English
Literature exclusion
Studies including < 20 immature permanent teeth
Studies with follow-up < 24 months
Case reports, case series
Animal studies
In vitro studies
Studies with incomplete outcome data (not defining both clinical and radiographic outcomes)
Review studies
Studies irrelevant to RET or CH/MTA or not peer-reviewed

RET studies: regenerative endodontic treatment

*CH* calcium hydroxide, *MTA* mineral trioxide aggregate

CH and MTA studies: studies employing apexification techniques (MTA: mineral trioxide apical plug or apical closure with CH)

#### Search strategy

To relate to the rationales of this review, the search strategy was based on the decision to exclude articles with short-term follow-up (< 2 years), articles with larger study samples with follow-ups of less than two years (*n* > 20, < 2 years), and articles with an intervention group less than 20 (*n* ≤ 20 cases). The search strategy was based on the decision to exclude studies with short follow-ups, larger study samples with < 2 years of follow-up, and intervention groups less than 20 (*n* ≤ 20 cases). A systematic screening of the literature included studies published between January 2004 and October 2019. To identify relevant studies, we systematically and thoroughly searched several databases of biomedical and dentistry literature. These databases were searched in accordance with the guidelines from the Swedish Agency for Assessment of Health Technology and Social Services (SBU 2017). Five electronic databases were used as information sources to retrieve relevant articles: PubMed, Web of Science, Cochrane Library, Ovid (Medline), and Embase. The rules of each database were respected, and the Booleans OR and AND were used to combine the Medical Subject Headings (MeSH), synonyms, and free terms according to the syntax rules of each database. The PRISMA Statement was endorsed (Moher et al. 2009). The search was not expanded within unpublished literature (registries or theses) (Table 2).

**Table 2:** Search block strategy

(Regenerative endodontic treatment OR regenerat* OR revital* OR endodontic regeneration OR regenerative endodontics OR pulp revascularization OR revasculari*)
AND
(immature permanent teeth OR open apex OR open apices OR root development OR pulp necrosis OR necrotic pulp OR apical periodontitis OR apical lesion OR apical abscess OR periapical*)
AND
(“dental trauma*” OR “tooth injuries” OR “traumatized immature*”)
AND
(“adverse*” OR “regenerat* failure*” OR “regenerat* healing)

#### Search terms

To identify eligible articles, we organised the search terms into blocks corresponding with the aim of this study. We received help and services from university librarians with expertise in literature review search strategies at Karolinska Institute library. Online Appendix 1 lists the exact combination of search terms from each database.

#### Study selection process

Three reviewers (AW, ML, and MES) performed blinded eligibility assessments. Only full-text articles were chosen for this review. The reviewers screened all the retrieved records by title and abstracts to reduce the risk of rejecting relevant reports (Edwards et al. 2002). The Rayyan QCRI program was used as an interface for eligibility assessment (https://rayyan.qcri.org). Disagreements between the reviewers were resolved by consensus. The assessment procedures were pilot tested, evaluated, and adjusted.

Finally, a prespecified data extraction form was developed for the data extraction process based on the Cochrane data extraction template. Efforts were made to screen for and identify duplicate publications by comparing clinical trial numbers, study sizes, treatment interventions, and names of the authors (Gotzsche 1989). Quality criteria regarding methodology implications, coherence, and adequacy of data were screened and comments and assessments were completed (Table 3). All retrieved articles were included regardless of their quality in order to cover and evaluate the scientific quality and reliability of the existing research: twelve studies were high quality, seven were medium quality, and two were low quality.

**Table 3:** Quality appraisal (Lewin et al. 2015)

References	Methodology and possible limitations	Coherence	Adequacy of data	Quality assessment
Ulusoy (2019)	Randomised clinical trial design. Reported predetermined power calculation of the sample size. Good explanation of inclusion/exclusion criteria. Strict definitions of outcomes. Traumatised necrotic incisors with apex opening > 1 mm. Random distribution in 4 scaffold groups: PRP; PRF; PP; BC. Irrigant: 1.25% NaOCl + 2% chlorhexidine + 17%EDTA. No instrumentation. Medication: TAB. Recall after 4 weeks. No collagen barrier. Materials: White MTA Pro Root, gj, composite Outcome measurements: clinical signs and symptoms; resolution of periapical disease; increase in root width; increase in root length; increase in radiographic root area; decrease in radiographic canal area; Sensitivity testing at baseline and follow ups. Periapical radiolucency at baseline and follow-up	The discussion and conclusions are supported by the results of the study, good transparency	Richness and quantity of data relevant and supporting the findings Good statistical methods Eligible for synthesis	Level of evidence: high
Chan (2016)	Longitudinal cohort study design. Predefined outcome measurements; Strict inclusion and exclusion criteria; The stage of root development was defined at baseline and follow-up; Periapical radiolucency at baseline and follow-up; Clinical signs and symptoms; Increase in root width. Increase in root length; Apical closure; Resolution of apical pathology; MTA placement from CEJ; MTA thickness and density; Sensitivity testing at baseline and follow ups; Periapical radiolucency at baseline and follow-up Limitations: control group not included	The discussion and conclusions are supported by the results of the study, good transparency	Richness and quantity of data relevant and supporting the findings Good statistical methods Eligible for synthesis	Level of evidence: moderate
Bücher (2016)	Retrospective case–control design. Long-term follow-up; sufficient study size; low drop-out; high clinical success; low prevalence of postoperative symptoms shows that the treatment is effective for survival. Predefined outcome measurements: Clinical signs and symptoms; root development stage; PAI index at baseline and follow-up; Quality of rf Limitations: No control group. Differences in treatment protocol. Pulpal diagnosis differed: irreversible pulpitis and pulp necrosis	The discussion and conclusions are supported by the results of the study, good transparency	Richness and quantity of data relevant and supporting the findings Good statistical methods Eligible for synthesis	Level of evidence: moderate
Ree (2017)	Retrospective cohort design. 83 immature teeth were treated over a 10-year period using MTA barrier and adhesive restorations Predefined outcome measurements: Clinical signs and symptoms; resolution of apical pathosis. Healed and non-healed Limitations: Differences in treatment protocol. Differences in inclusion: In 44 cases, previous root canal treatment was performed, in the rest pulp necrosis, dens invaginatus and no previous root canal treatment. No control group	The discussion and conclusions are supported by the results of the study, good transparency	Richness and quantity of data relevant and supporting the findings Good statistical methods Eligible for synthesis	Level of evidence: moderate
Demiriz (2017)	Retrospective cohort design. Two group settings: experimental group with extrusion of MTA and control group with no extrusion of MTA. Outcome measurements: clinical signs and symptoms; periapical healing; measurement of the amount of extruded MTA on radiographs. Radiographic follow-up of the amount of extruded MTA over time. Included control group Limitations: The stage of root development was not defined at baseline or follow-up. Pulpal diagnosis not defined at baseline. Reason for treatment not defined	The discussion and conclusions are supported by the results of the study, good transparency	Richness and quantity of data relevant and supporting the findings Good statistical methods Eligible for synthesis	Level of evidence: moderate
Kandemir Demirci (2014)	Randomised clinical trial design. Long-term follow-up; big enough study size; low drop-out; high clinical success; low prevalence of postoperative symptoms shows that the treatment is effective for survival. Predefined outcome measurements: Authors blinded for the purpose of radiographical interpretation; Healed—asymptomatic, radiograph showed PAI 1 or 2; Healing – asymptomatic, radiograph showed PAI 3 or 4, with score improved at follow-up from immediate post-treatment radiograph; Not healed—either symptomatic or asymptomatic, but the radiograph revealed no decrease or an increase in the size of the preexisting radiolucency at follow-up from immediate post-treatment radiograph (PAI 3–5)	The discussion and conclusions are supported by the results of the study, good transparency	Richness and quantity of data relevant and supporting the findings Good statistical methods Eligible for synthesis	Level of evidence: high
Mente (2013)	Retrospective longitudinal cohort design. Long-term follow-up; sufficient study size; low drop-out; high clinical success; low prevalence of postoperative symptoms shows that the treatment is effective for survival. Predefined outcome measurements: The presence of preoperative apical periodontitis was identified as the most important prognostic factor, and success rates remained consistently high, even after follow-up of more than 4 years Limitations: The stage of root development was not defined at baseline or follow-up. No control group included	The discussion and conclusions are supported by the results of the study, good transparency	Richness and quantity of data relevant and supporting the findings Good statistical methods Eligible for synthesis	Level of evidence: moderate

## Results

**Fig. 1:**
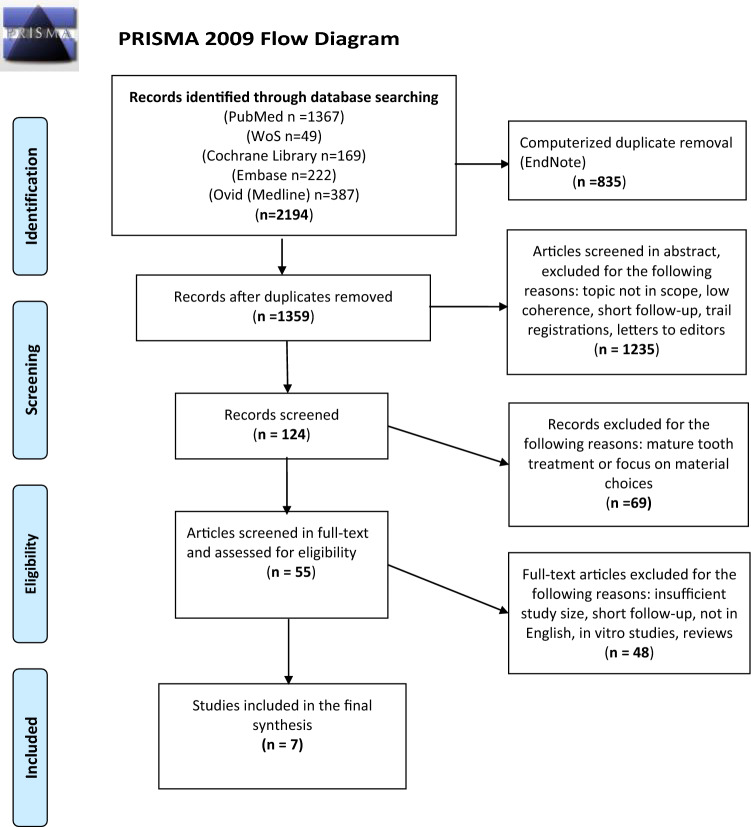
PRISMA 2009 flow diagram

After removing duplicates, the database search resulted in 1359 hits (Fig. 1). After the articles were screened by title and abstract, 55 were selected to be read in full. Of these, 48 were excluded for various reasons (Table 4). Finally, seven articles were considered eligible for inclusion in the final qualitative synthesis (two in the regenerative treatment group and five in the MTA treatment group) (Tables 5 and 6). Further attempts were made to identify on-going studies that may be eligible for inclusion. As suggested by Ghough et al. (2017), the authors of three studies that matched the inclusion criteria with the exception of follow-up length were sent an e-mail that asked them whether they had performed a 24-month follow-up since publication (Bezgin et al. 2015; Nagata et al. 2014; Gough and Thomas 2017). Although one of the authors confirmed that he had performed a 24-month follow-up with controls, the study was excluded because of the study size (Nagy et al. 2014). We excluded the vast majority of the screened records in the RET group because of small study size and insufficient follow-up (3–12 months).

**Table 4:** Excluded RET and MTA studies and reasons for exclusion

Reference	Title	Reason for exclusion
Nagmode et al. (2016)	The effect of mineral trioxide aggregate on the periapical tissues after unintentional extrusion beyond the apical foramen. Case Rep Dent 2016; 2016:3590680	Too small study size (*n* = 3)
Raldi et al. (2009)	Treatment options for teeth with open apices and apical periodontitis. J Can Dent Assoc 2009; 75:591–6	Too small study size (*n* = 1)
Vanka et al. (2010)	Apexification with MTA using internal matrix: report of 2 cases. J Clin Pediatr Dent 2010; 34:197–200	Too small study size (*n* = 2)
Tahan et al. (2010)	Effect of unintentionally extruded mineral trioxide aggregate in treatment of tooth with periradicular lesion: a case report. J Endod 2010; 36:760–3	Too small study size (*n* = 1)
Giovarruscio et al. (2013)	A technique for placement of apical MTA plugs using modified Thermafil carriers for the filling of canals with wide apices. Int Endod J 2013; 46:88–97	Too small study size (*n* = 1)
Chung et al. (2011)	An interesting healing outcome of a replanted immature permanent tooth: a case report. Dent Traumatol 2011; 27:77–80	Too small study size (*n* = 1)
Mohammadi and Yazdizadeh (2011)	Obturation of immature non-vital tooth using MTA. Case report. NY State Dent J 2011; 77:33–5	Too small study size (*n* = 1)
Maturo et al. (2009)	MTA applications in pediatric dentistry. Oral Implantol (Rome) 2009; 2:37–44	Too small study size (*n* = 1)
Asgary et al. 2016	Regenerative endodontic treatment versus apical plug in immature teeth: three-year follow-up. J Clin Pediatr Dent 2016; 40:356–60	Too small study size (*n* = 1)
Wang et al. 2015	Pulp revascularization on permanent teeth with open apices in a middle-aged patient. J Endod 2015; 41:1571–5	Too small study size (*n* = 2)
McCabe (2015)	Revascularization of an immature tooth with apical periodontitis using a single visit protocol: a case report. Int Endod J 2015; 48:484–97	Too small study size (n = 1)
Dudeja (2015)	Pulp revascularization- it’s your future whether you know it or not? J Clin Diagn Res 2015; 9:ZR01–4	Too small study size (n = 5)
Nagata et al. 2014	Traumatized immature teeth treated with 2 protocols of pulp revascularization. J Endod 2014; 40:606–12	Too short follow-up period
Becerra et al. (2014)	Histologic study of a human immature permanent premolar with chronic apical abscess after revascularization/revitalization J Endod 2014; 40:133–9	Too small study size (n = 1)
Mishra et al. (2013)	Platelet-rich fibrin-mediated revitalization of immature necrotic tooth. Contemp Clin Dent 2013; 4:412–5	Too small study size (n = 1)
Badole et al. (2015)	Nonsurgical endodontic treatment of permanent maxillary incisors with immature apex and a large periapical lesion: a case report. Gen Dent 2015; 63:58–60	Too small study size (n = 1)
Al Ansary et al. (2009)	Interventions for treating traumatized necrotic immature permanent anterior teeth: including a calcific barrier & root strengthening. Dent traumatology, Vol 25; Issue 4; p 367–379	Review. Not an intervention study
Damle et al. (2012)	Apexification of anterior teeth: a comparative evaluation of mineral trioxide aggregate and calcium hydroxide paste. J of clinical pediatric dentistry, Vol 36; Issue 3; p 263–268	Too short follow-up (12 months)
Estefan et al. (2016)	Influence of age and apical diameter on the success of endodontic regeneration procedures. J Endod 2016; 42:1620–5	Too short follow-up (12 months)
Saoud et al. (2014)	Clinical and radiographic outcomes of traumatized immature permanent necrotic teeth after revascularization/revitalization therapy. J Endod 2014; 40:1946–52	Too short follow-up (12 months)
Nie (2014)	Apexification of abnormal central cusp teeth with four kinds of calcium hydroxide preparations. Chinese journal of tissue engineering research; Vol 17; Issue 8; p 1398–1403	Article not in English
Simon et al. (2007)	The use of mineral trioxide aggregate in one-visit apexification treatment: a prospective study. Int Endod J; Vol 40; Issue 3; p 186–197	Too short follow-up (12 months)
Chen et al. (2015)	Potential dental pulp revascularization and odonto-/osteogenic capacity of a novel transplant combined with dental pulp stem cells and platelet-rich fibrin. Cell and tissue research; Vol 361; Issue 2; p 439–455	In vitro animal model
Ragab et al. (2019)	Comparative study between revitalization of necrotic immature permanent anterior teeth with and without platelet rich fibrin: a randomized controlled trial. J of clinical ped dent. Vol 43; Issue 2; p 78–85	Too short follow-up (12 months)
Shivashankar et al. (2017)	Comparison of the effect of PRP, PRF and induced bleeding in the revascularization of teeth with necrotic pulpa and open apex: a triple blind randomized trial. J of clinical and diagnostic research; Vol 11: Issue 6; p Zc34-zc39	Too short follow-up (12 months)
Medina-Fernandez et al. (2019)	Acellular biomaterial strategies for endodontic regeneration. J biomaterials science; Vol 7; Issue 2; p 506–509	Review. Not an intervention study
EzEldeen et al. (2015)	3-dimensional analysis of regenerative endodontic treatment outcome. J Endod 2015; 41:317–24	Too small study size (*n* = 5). Too short follow-up (12 months)
Kahler et al. (2014)	Revascularization outcomes: a prospective analysis of 16 consecutive cases. J of endod, Vol 40; Issue 3; p 333–8	Too small study size (*n* = 13). Too short follow-up (18 months)
Kaushik et al. (2016)	Biomimetic microenvironments for regenerative endodontics. Biomaterials research; Vol 20; Issue 0; p 14	Not an intervention study
Kumar et al. (2014)	Management of 2 teeth diagnosed with dens invaginatus with regenerative endodontics and apexification in the same patient: a case report and review. J Endod 2014; 40:725–31	Too small study size (*n* = 1)
Shah et al. (2008)	Efficacy of revascularization to induce apexification/apexogensis in infected, nonvital, immature teeth: a pilot clinical study. J Endod 2008; 34:919–25	Too small study size (*n* = 14)
El-Meligy and Avery (2006)	Comparison of apexification with mineral trioxide aggregate and calcium hydroxide. Pediatr Dent 2006; 28:248–53	Too small study size (*n* = 15)
Lin et al. 2017	Regenerative endodontics versus apexification in immature permanent teeth with apical periodontitis: A prospective randomized controlled study. JOE; 43(11): 1821–1827	Too short follow-up (12 months)
Nazzal et al. (2018)	A prospective clinical study of regenerative endodontic treatment of traumatized immature teeth with necrotic pulps using bi antibiotic paste. International endodontic journal. Vol.51 Suppl 3, p.e204-e21	Too small study size (*n* = 15)
McTigue et al. 2013	Management of Immature Permanent Teeth With Pulpal Necrosis: A Case Series. Pediatric dentistry. 2013; 35(1):55–60	Too small study size at follow-up
Chueh et al. (2009)	Regenerative endodontic treatment for necrotic immature permanent teeth. Journal of endodontics. 2009;35(2):160–4	Too small study size at follow-up
Chen et al. (2012)	Responses of immature permanent teeth with infected necrotic pulp tissue and apical periodontitis/abscess to revascularization procedures. International endodontic journal. 2012; 45(3):294–305	Too small study size at follow-up
Jeeruphan et al. (2012)	Mahidol study 1: comparison of radiographic and survival outcomes of immature teeth treated with either regenerative endodontic or apexification methods: a retrospective study. Journal of endodontics. 2012;38(10):1330–6	Too short follow-up in the RET group
Nagy et al. (2014)	Regenerative potential of immature permanent teeth with necrotic pulps after different regenerative protocols. Journal of endodontics. 2014; 40(2):192–8	Too small study size at follow-up
Bose et al. (2009)	Dental Pulp Revascularization of Necrotic Permanent Teeth with Immature Apices. The Journal of clinical pediatric dentistry. 2016;40(5):361–6	Too small study size at follow-up
lobaid et al. (2014)	Radiographic and clinical outcomes of the treatment of immature permanent teeth by revascularization or apexification: a pilot retrospective cohort study. Journal of endodontics. 2014;40(8):1063–70	Too small study size at follow-up
El Ashiry et al. (2016)	Dental Pulp Revascularization of Necrotic Permanent Teeth with Immature Apices. The Journal of clinical pediatric dentistry. 2016;40(5):361–6	Too small study size at follow-up
Silujjai et al. (2017)	Treatment Outcomes of Apexification or Revascularization in Nonvital Immature Permanent Teeth: A Retrospective Study. Journal of endodontics. 2017;43(2):238–45	Too small study size at follow-up
Bukhari et al. (2016)	Outcome of Revascularization Procedure: A Retrospective Case Series. Journal of endodontics. 2016; 42(12):1752–9	Too small study size at follow-up
Moore et al. (2011)	Treatment of open apex teeth using two types of white mineral trioxide aggregate after initial dressing with calcium hydroxide in children. Dental traumatology: official publication of International Association for Dental Traumatology. 2011;27(3):166–73	Too short follow-up
Witherspoon et al. (2008)	Retrospective analysis of open apex teeth obturated with mineral trioxide aggregate. Journal of endodontics. 2008; 34(10):1171–6	Follow up periods not stated
Plascencia et al. (2017)	Management of permanent teeth with necrotic pulps and open apices according to the stage of root development. Journal of clinical and experimental dentistry. 2017;9(11):e1329-e39	Follow up periods not stated
Kleier and Barr (1991)	A study of endodontically apexified teeth. Endodontics & dental traumatology. 1991;7(3):112–7	Follow up periods not stated

**Table 5:** RET study characteristics

Author year	Study design	Size	Tooth type	Age (y) mean	Aetiology	TG	Irrigant	DRE	DRE, weeks	Scaff-old	Barrier	Outcome	Follow-up, months	Drop- out
Ulusoy (2019)	RCT	88	100%A	8–11	100% T	I = 66; C = 22	1.25% NaOCl; 2% CHX; 17% EDTA	TAP (20 mg Clinda, 20 mg Cipro, 20 mg Metro)	4	PRF; PRP; PP; BC	WMTA	100% AP-healing; 82% PP C-apexification 76% BC C-apexification 71% PRF C-apexification 67% PRP C-apexification 4% O-apexification; 22% No IRL/IRT; 86% Positive PST	28.25 ± 1.2	*n* = 12
Chan (2016)	LCS	28	82% A 10% P 8% M	9	79% T 36% DA 7% DC	I = 28; C = 0	5.25% NaOCl	TAP (100 mg Cefa, 500 mg Cipro, 500 mg Metro)	2–6	BC	CB + WMTA	100% AP-healing; 96% survival, 93% clinical success; 92% IRL; 82% IRT; 31% C-apexification; 54% P-apexification; 15% N-apexification; 100% Negative PST	30 ± 12,7	*n* = 2

*RCT* randomised clinical trial, *LCS* longitudinal cohort study, *N* study size, *A* anterior tooth, *P* premolar, *M* molar, *T* trauma, *DA* dental anomalies, *DC* dental caries, *TG* treatment groups, *PRP* platelet rich plasma, *PRF *platelet rich fibrin, *PP* platelet pellet, *DRE* root canal dressing, *BC* induced blood clot, *CH* calcium hydroxide, *TAP* triple antibiotic paste, *CHX* chlorhexidine gluconate, *I* intervention group, *C* control group, *WMTA* white MTA Pro Root, CB collagen barrier: Clinda-clindamycin: Cipro-ciprofloxacin, *Metro* metronidazole, *Cefa* cefaclor, *AP* healing-radiographic resolution of apical pathology, *Positive PST* positive response to pulp sensitivity tests, *Negative PST* negative response to pulp sensitivity tests, *IRL* increase in root length, *IRT* increase in root thickness, *C-apexification* complete apical closure, *P-apexification* partial apical closure, *N-apexification* no apical closure, *O-apexification* ongoing apical closure

**Table 6:** MTA study characteristics

Author (year)	Study design	Size	Tooth type	Age (y) mean	Aetiology	TG	Irrigant	DRE	DRE, weeks	RF material	Outcome	Follow -p, months	Drop- outs	Failure
Bucher (2016)	RECO	98	A P M	> 6	66.3% T 1% DC 7.1% RETR 7.1% MIH	I = 98; C = 0	3% NaOCl; 24% EDTA; 2% CHX; 0.9% NaCl	CH/L	1–4	WMTA/GP	97% functional retention; 94% clinical success; 71.4% optimal root filling; 3.1% failure; 90.2% AP-healing; 95% survival trauma; 70% survival non-trauma	30	N.S	*n* = 3
Ree (2017)	RECO	83	A	10.15	71% T 4% DA 25% RETR	I = 20; C = 0	5% NaOCl; 17%EDTA	CH	3–4	CS/WMTA/GP/R/FP	96% AP-healing 98.5% survival	100	*n* = 14	*n* = 1
Demiriz (2017)	RECO	55	A	9.47	NS	I:MTA extrusion = 21 C:MTA no extrusion = 34	2.5%NaOCl	CH	N.S	GMTA	100% survival 90,4% MTA extrusion complete AP-healing; 100% control group with no MTA extrusion complete AP-healing 89,5% reduced amount of extruded MTA in teeth with complete healing 10% extruded MTA absent after 3 years	36	*n* = 10	N.S
Kandemir Demirci (2014)	RCT	90	A	18–40; mean 23.34	T DC DA	I(MTA) = 45; C(CH) = 45	2.5%NaOCl; 17% EDTA; 2% CHX	CH	MTA1;CH N.S	WMTA/GP	MTA: 92% survival; 8% failed; 74% apexification /AP-healing; 18% ongoing AP-healing, 8% no AP-healing CH: 91% AP-healing / 79% complete AP-healing; 12% apexification / ongoing AP-healing; 9% no AP-healing	mean 32.3	*n* = 17; (87% for MTA and 76% for CH)	*n* = 3
Mente (2013)	RELCO	221	A P M	9–80 (median 43)	N.S	*I* = 252; *C* = 0	NaOCl EDTA	CH	N.S:	WMTA/GP	96% survival 90% AP-healing Prognostic factors: 2. Experience of TR-provider 3. Number of treatment sessions 4. MTA extrusion	12–128 (median 21)	*n* = 27	N.S

*RECO* retrospective cohort study, *RCT* randomised clinical trial, *RELCO* retrospective longitudinal cohort study, *A* anterior, *P* premolar, *M* molar, *T* trauma, *DC* dental caries, *DA* dental anomalies, *RETR* endodontic retreatment cases, *MIH* hypofosfatasia, *DRE* root canal dressing, *CH* calcium hydroxide, *CHX* chloridehexidine gluconate, *I* intervention group, *C* control group, *NS* not stated, RF material root-filling material, *WMTA* white MTA Pro Root, *GMTA* grey MTA Angelus, *GP* gutta percha, *CS* calcium sulfate, *R* resilon, *FP* fiber post, *L* Corticosteroid Ledermix, *TG* treatment groups, *AP* healing-radiographic resolution of apical pathology, *TR-provider* treatment provider

### Descriptive findings

The results showed that the included studies in both groups were heterogeneous regarding the outcome measurements. Only three studies reported Cvek’s stage of root development at baseline—one study in the RET group (Chan et al. 2017) and two studies in the MTA group (Kandemir Demirci et al. 2019; Bucher et al. 2016). Based on the comparison of the mean age values between the two study groups, we assumed that RET studies included teeth with a lower stage of root development than the MTA studies (mean age RET 8.33 years; mean age MTA 18.39 years) (Table 7). As the average study size of the two included RET studies was small (28 in one and 88 in one divided into four treatment groups including 22 cases each) (Table 3), the differences in the intervention effects might have been overlooked or misinterpreted (Gluud 2006).

**Table 7:** Main findings in the included MTA and RET studies

	RET studies	MTA studies
Patient mean age	8.33 years	18.39 years
Most prevalent aetiology	Trauma	Trauma
Most prevalent treated tooth type	Anterior	Anterior
Total treated cases	116	578
Range of resolution of apical pathology	100%	90–100%
Mean follow-up (months)	29 months	221 months

### Comparison between the included MTA and RET studies

The outcome measures between the MTA and RET studies were not identical as different treatment modalities were the subject of evaluations. To address the aim of this study (What measures were used to assess the treatment outcomes of regenerative endodontic procedures and apexification techniques with calcium hydroxide or MTA/gutta percha), the following measurements were made in both study groups: healing of apical periodontitis, survival, success, failure, functional retention, adverse effects, and apical closure (apexification).

In the RET study group, the deviant outcome measures were root lengthening and root thickening, which basically refer to continued root development. In the MTA group, the deviant outcome measure was the quality of root filling. This finding makes it difficult to perform a complete statistical comparison of the outcomes between the two groups. If the studies were more homogenous, the results could have been combined in a meta-analysis with a goal of increasing statistical significance.

Although the studies included in this review revealed different disinfecting routines, the treatment protocols were essentially homogenous: disinfection, intra-canal antimicrobial dressing, and blood clot as a matrix for regeneration. Several treatment protocols were used and are addressed in detail in the discussion (Alobaid et al. 2014; Chen et al. 2012). Varying concentrations of irrigation with NaOCl were used in the majority of the studies, but no significant difference in the overall outcomes was reported. The same observation concerns the root canal dressings. The disinfecting agent triple antibiotic paste (TAP) was used in both of the RET studies. The Hoshino`s classic mixture of triple antibiotic paste (TAP) (Hoshino et al. 1996) was modified and applied (mTAP) (Thibodeau and Trope 2007). Calcium hydroxide was used as a disinfecting agent in the MTA study group. Even in this group, no significant difference in overall outcomes was observed between the studies. The methods for inducing an apical barrier were compared, apexification was compared to apical plug techniques, and both techniques were compared to no apexification treatment (conventional root canal obturation with gutta percha).

### Study design

Only two of the included studies were randomised clinical trials—one of the RET studies (Ulusoy et al. 2019) and one of the MTA study group (Kandemir Demirci et al. 2019)—and the majority of the studies lacked control groups. Therefore, we could not use statistical meta-analysis models to estimate the effect size (Dixon-Woods et al. 2006).

### Pulpal diagnosis

The qualitative analyses of the included cases showed that the majority of the treated teeth were diagnosed with pulp necrosis. In the RET study group, the most common basis of diagnosing pulp necrosis was preoperative negative response to cold testing and electric pulp tests. Both of the RET studies were designed to record pulp sensitivity testing both preoperatively and postoperatively. The results were inconclusive as the first study showed that 86% of the regenerated cases responded positively to sensitivity tests at follow-up (Ulusoy et al. 2019). The second study showed that 100% of the regenerated cases did not respond positively to sensitivity tests at follow-up (Chan et al. 2017). In the MTA study group, the majority of the included studies did not state accurate information about pulp sensitivity before treatment. That is, four of the five included MTA studies were retrospective so the data for sensitivity testing before treatment might have been unavailable. Standardised examination methods, including sensitivity testing at baseline, were used in a MTA study with a RCT design (Kandemir Demirci et al. 2019). In fact, the retreatment cases and cases with irreversible pulpitis were also included in the MTA treatment group (Ree and Schwartz 2017).

### Tooth type

Anterior teeth were the most common subject for treatment in both groups (see Tables 7, 8).

**Table 8:** Outcome measures for the RET and MTA included studies

Outcome measures RET group	Outcome measures MTA group
Healing of apical periodontitis (radiographic radiolucency)	Healing of apical periodontitis (radiographic radiolucency)
Survival, Success, Failure	Survival, Success, Failure
Continued root development: Root wall lengthening Root wall thickening	Quality of root filling/MTA placement in relation to apex Overfilled Underfilled
Functional retention	Functional retention
Adverse effects	Adverse effects
Apical closure (apexification)	Apical closure (apexification)

### Aetiology

Traumatic dental injuries leading to pulp necrosis and apical periodontitis were the most common reason for treatment in both RET and MTA study groups. Exhaustive data about the specific type of trauma injury were reported in five of the included studies (Bucher et al. 2016; Ree and Schwartz 2017; Kandemir Demirci et al. 2019; Chan et al. 2017; Ulusoy et al. 2019), and two studies did not state the cause for the treatment (Demiriz and Hazar 2017; Mente et al. 2013). In both study groups, the overall trauma frequency varied between 46 and 100% (Table 9).

**Table 9:** Trauma as a cause for treatment in RET and MTA studies

Author	Reported type of trauma	Frequency per type of trauma, %	Total trauma frequency, %
Bucher	Crown fracture	46	46
	Luxation	20	
Ulusoy	Complicated crown fracture	100	100
Chan	Avulsion, intrusion	21	78
	Luxation, concussion	21	
	Crown fracture	36	
Ree	Uncomplicated crown or root fracture	16	71
	(Sub)luxation	31	
	Horizontal root fracture	6	
	Avulsion	11	
	Intrusion	7	
	Combination trauma	25	
Kandemir Demirci	Crown fracture	69	69
Demiriz	Not Stated	Not Stated	Not Stated
Mente	Not Stated	Not Stated	Not Stated

### Clinical treatment protocols

#### Irrigants

All of the included studies described the treatment procedures exhaustively. Several protocol deviations were detected, but these did not influence the reported overall outcome and survival rates. All the studies used sodium hypochlorite (NaOCl) at various concentrations as the main irrigant. Chlorhexidine gluconate (CHX) was used as supplemental irrigation in 50% of the RET studies and 20% of the MTA studies. EDTA was used in 50% of the RET studies and in 80% of the MTA studies.

#### Intra-canal medication

All studies included in the MTA group used calcium hydroxide (CH) as a canal medicament. In one MTA study, four severe trauma cases were treated with calcium hydroxide in combination with other antibacterial medicament (Corticosteroids, Ledermix) (Bucher et al. 2016). In the RET study group, the intra-canal medication consisted of two combinations of triple antibiotics: 20-mg clindamycin, 20-mg ciprofloxacin, and 20-mg metronidazole (Ulusoy et al. 2019) and 100-mg cefaclor, 500-mg ciprofloxacin, and 500-mg metronidazole (Chan et al. 2017).

#### Total time of medication

In the RET group, one of the studies used intra-canal medication for 4 weeks (Ulusoy et al. 2019), and one study used the intra-canal medication between 2 and 6 weeks (Chan et al. 2017). In the MTA study group, three of the included studies reported similar time of medication—i.e. one to four weeks—with the exception of a prolonged time frame in one of the control groups where the final treatment was postponed until a complete apical calcific barrier was observed radiographically and confirmed clinically (Kandemir Demirci et al. 2019). Two of the MTA studies did not provide data about the total medication time (Demiriz and Hazar 2017; Mente et al. 2013).

#### Clinical regenerative procedures in RET studies

The two studies included in the RET study group differed from each other with respect to the regenerative protocols. In the first study (Ulusoy et al. 2019), treatments were divided into four groups. In three of the groups, the patient’s blood was centrifuged and different platelet concentrates were introduced into the root canal without prior induction of bleeding from the periapical tissues. In the fourth treatment group in the same study and in the second treatment group in another study (Chan et al. 2017), the clinical protocol included induction of bleeding from the periapical tissues to allow the formation of a blood clot. Both methods effectively promoted apical closure and continuation of the root maturation and no significant differences in outcomes were observed.

#### Scaffold in RET studies

A collagen scaffold to aid the placement of MTA over the blood clot was used in one of the included studies (Chan et al. 2017), although no such scaffold was used in the other study (Ulusoy et al. 2019).

#### Changes in root dimensions

Both of the included RET studies reported accurate information on continued root development and used the same system to measure changes in root dimensions. Preoperative and postoperative radiographs were compared with the help of ImageJ software and TurboReg plug-in. This method minimises different angulations and corrects minor distortions (Bose et al. 2009). The results from Chan et al. (2017) show that the root thickness in the apical area and root lengthening increased the most in teeth with Cvek’s root development stages 1 and 2 compared to teeth with root development stages 3 and 4. In Ulusoy’s data, we could not find a correlation between root development stage and changes in root dimensions due to the study’s focus on evaluation of different scaffolds (Ulusoy et al. 2019). In addition, no statistical significance could be calculated between the different groups as similar increases in root dimensions were evident in all groups regardless the type of scaffold applied. None of the included studies in the MTA group reported increases in root dimensions.

#### The mean follow-up time

The RET and MTA study group’s mean time for follow-up was 29 months and 221 months, respectively.

#### Treatment complication

Crown discoloration after application of MTA was reported at follow-up in both study groups. Other reported complications were presence of persistent apical periodontitis, clinical symptoms, root resorption, and root fracture (Table 10).

**Table 10:** Reported complications in RET and MTA studies

Author	*N*	Nature of complication at follow-up
Ulusoy (2019)	2	Spontaneous pain and extreme sensitivity to percussion
Chan (2016)	16	MTA discoloration
	2	Recurrence of symptoms
Bucher (2016)	19	MTA discoloration
	2	Inflammatory root resorption
	1	Root fracture
Ree (2017)	34	MTA discoloration
	3	Persistent apical periodontitis
	3	Invasive cervical resorption
	1	Replacement root resorption
	1	Horizontal root fracture
Demiriz (2017)	1	Persistent apical periodontitis
	1	Inflammatory root resorption
	1	Incomplete healing
Mente (2013)	6	Persisting symptoms
	3	Persistent apical periodontitis
Kandemir Demirci (2014)	6	Persistent apical periodontitis with clinical symptoms

### Success and survival

An important finding concerning both groups was the reported high rates of success and survival treatment (90–96%). Surprisingly, the presence of trauma did not seem to negatively influence the outcome of the treatment.

### Drop-out rates

The overall drop-out rates were low (between 7 and 19%). The most common stated reason for the drop-out was missing follow-up appointments (patients refused to participate or patients could not be contacted). Several other less common reasons were also reported: choosing extraction instead of further treatment because of financial reasons, patients not returning for the final treatment, and the lack of retrievable radiographs in the studies with retrospective design.

## Discussion

Throughout the review, we assessed the review methods for transparency of literature searches, appropriateness and relevance to the research question, and the nature and evidence of the individual studies. We concluded that the methods and studies were relevant, the findings were appropriately interpreted (Gough and Thomas 2017), and the review design and methods addressed the research questions.

Checklists and flow diagrams were used for clear recording from searching to screening (Moher et al. 2009; SBU 2017). Critical appraisal was adopted to examine the trustworthiness, value, and relevance of the included studies (DH 1996; Oakley 1998). A CASP checklist from the Critical Appraisal Programme, adapted from Guyatt et al. (1994), was used to evaluate the overall study design (Guyatt et al. 1994).

### Search strategy

Before this study started, a search in Cochrane Library and PROSPERO was conducted to map all published and in progress reviews. The whole search process was transparently described and defined with the help of a librarian from Karolinska Institute.

As the search question and search strategy defined, we identified the best matching terms, synonyms, controlled vocabularies (MeSH terms), Boolean operators, and truncations according to the ‘golden-standard’ article for mapping and extraction of appropriate terms (Nicoloso et al. 2019). After a pilot search was conducted to explore the sensitivity and specificity of the search, the field codes were defined more precisely. Advanced search functions were used in appropriate blocks corresponding to the aims of the review. As dentistry is an integrated part of the medical topic, we searched PubMed before adjusting search strategies for additional databases. These databases were scoped for suitability. Web of Science, Medline (Ovid), Cochrane Library, and Embase provided an exhaustive coverage of the literature in the medical field (Schlosser et al. 2007). All databases were practical and offered numerous hits. For example, Medline (Ovid) provided a more expanded spectrum of journals than PubMed, (E-pub Ahead of Print and In-Process). However, the searches in Web of Science, which is a multidisciplinary database, resulted in fewer hits (*n* = 49). This result could be explained by the way the citation analysis was designed: it was intended to satisfy the needs of users of citation analysis, a field discussed and debated by scientists for decades.

As five electronic databases were searched, the risk for database biases was minimised according to AMSTAR (Perry et al. 2017; Shea et al. 2017). Several authors and the Cochrane Collaboration state that multiple appropriate databases should be chosen so that the yield of relevant studies is maximised and the risk for database bias is minimised (Moher et al. 2009; Schlosser et al. 2007). In this review, however, we found that many of the retrieved studies were duplicates (approximately 40%), evidence that the broad search strategy had high specificity.

When it comes to the specific terms, it is worth clarifying that the terms ‘traumatised’ and ‘immature permanent teeth’ were chosen for the pilot search, a strategy that produced few results. Therefore, these terms were not added in the final search as the final search was prioritised to include ‘all immature permanent teeth’. Because the search was conducted recently and the author of this report receives weekly updates with RSS and alerts, it was decided that no ‘new’ search was needed. No grey literature search was conducted with regard to the inclusion criterium only published peer-reviewed articles (the background, objective, aim, eligibility criteria, method, and outcomes). To summarise, the search strategy was well targeted and successful, resulting in 21 matching hits.

### Screening and classification

Three authors conducted the screenings independently and blinded. The authors screened the articles by title and abstract and read the full text of the potentially eligible articles. A majority rule was adopted in the decision-making with respect to the eligibility of the studies. A fourth participant, the principal investigator with experience in systematic reviews, was consulted for a final decision about eligibility, discrepancies, and classification with regard to the inclusion and exclusion criteria applied in the process. Consequently, the fact that four persons were involved assures good structure and minimises the risk for source selection bias (Schlosser et al. 2007). The excluded articles were classified to assure transparency in the selection process (Table 5).

### Quality assessment

As Schlosser et al. state, ‘a systematic review can only be as sound as the included studies’; therefore, we find it very important to provide quality assessment as studies might have a different quality level (Schlosser et al. 2007). Several quality assessments suitable for the included studies were considered: the STROBE checklist consisting of 22 questions (STROBE Statement-checklist of items that should be included in reports of observational studies) and the CONSORT statement (SBU 2017). As the included studies had different study designs, we adopted the modified quality assessment developed by Lewin et al. (2015) (GRADE-CERQual) (Lewin et al. 2015). An appraisal of the research question was made to assess the correspondence with the results. Moreover, evaluation of interventions, possible limitations, adequacy of data, statistical methods, and conclusions were made to strengthen the scientific evidence of the findings.

### Risk of bias assessment

Two different tools for assessing risk of bias were used: ROBINS-I tool (for assessing risk of bias in non-randomised studies of interventions) (Sterne et al. 2016) and RoB 2.0 tool (The Cochrane Collaboration’s tool for assessing risk of bias in RCTs) (Higgins et al. 2011). In accordance with the tools, the possible risk for bias in the included studies was organised and evaluated in different domains (i.e. random sequence generation and allocation concealment, blinding of participants and personnel regarding the treatment choice or the medicament choice, blinding of outcome data interpretation, and selective outcome data reporting). In particular, the lack of an adequate allocation sequence generation method is possible source of selection bias; that is, there is an uneven distribution between the different treatment groups that could result in overestimation of the treatment effect. Furthermore, the rating of several studies was challenging because blinding was not always feasible to personnel and participants and only one study reported predetermined power calculation of the sample size (Ulusoy et al. 2019). Online Appendix 2 specifies the domain questions considered during bias assessment with the ROBINS-I tool (see Tables 11, 12).

**Table 11:** Cochrane Collaboration’s tool for assessing risk of bias RoB 2.0 for RCT studies

Bias domain	Source of bias	Support for judgment	Review authors’ judgment (low, unclear or high risk of bias)	
			R1: Ulusoy et al (2019)	R2: Kandemir Demirci et al (2019)
Selection bias	Random sequence generation	The allocation sequence was random. No baseline difference between the intervention groups could be detected	**Low**	**Low**
	Allocation concealment		**Low**	**Low**
Performance bias	Blinding of participants and personnel	R1: Blinding was not possible for obvious reasons R2: blinded participants but not personnel	*High*	***Unclear***
Detection bias	Blinding of outcome assessment	R1: Outcome assessors aware of the intervention received by study participants. Unclear if outcome assessment was influenced by knowledge of intervention received R2: radiological assessment by two independent blinded investigators	***Unclear***	**Low**
Attrition bias	Incomplete outcome data	R1 and R2: Data available for all, or nearly all, participants randomised	**Low**	**Low**
Reporting bias	Selective reporting	R1 and R2: no information if results were analysed in accordance with a prespecified analysis plan that was finalised before unblinded outcome data were available for analysis	***Unclear***	***Unclear***
Overall bias			***Some concerns***	***Some concerns***

**Table 12:**
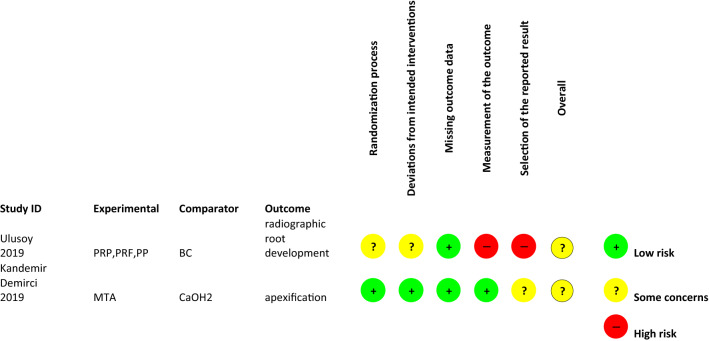
Risk of bias assessment RoB2 tool: RCT studies with intention-to-treat

### Study characteristics

Subject characteristics such as age, sex, aetiology, and tooth type were presented thoroughly in all studies in both groups. The majority of the studies had a complete and convincing reporting of the outcome data. Efforts were made to apply descriptive statistical methods. The studies in the MTA group had a low drop-out rate, included a large amount of teeth, and had a follow-up period longer than the RET group.

### Treatment protocols

#### Irrigation

With well-documented effects, NaOCl is the golden standard for disinfection in endodontics. The vast majority of the included studies used sodium hypochlorite (NaOCl) as the main irrigant. NaOCl concentrations varied between 1.25 and 5.25%. NaOCl has been used solely or in combination with EDTA, CHX, or NaCl. Several studies indicate that regular irrigation exchange and the use of large volumes of irrigating solution are essential for securing optimal antibacterial action (Siqueira et al. 2000). Nevertheless, none of the included studies reported standardised irrigation volumes as suggested in a consensus report from the European Society of Endodontology (Endodontology 2006; Galler et al. 2016). Another important issue is the discussion about the optimal concentration of NaOCl in regenerative procedures. It has been speculated that the higher the concentration used, the greater the risk for toxic effect on the stem cells of the apical papilla and the risk for damage (SCAP: s) (Martin et al. 2014). This problem could be avoided if the NaOCl concentration is reduced. Byström et al., in a classic study, showed that there was no difference between the antibacterial effect of 0.5% and 5% NaOCl solutions (Bystrom and Sundqvist 1985). Moreover, the combined use of NaOCl and EDTA is more efficient than NaOCl alone as EDTA can dissolve the smear layer.

Several endodontic studies have found that the supplemental use of passive ultrasonic irrigation increases the antibacterial efficacy of root canal irrigants, which is particularly important when minimal or no mechanical instrumentation of the root canal is recommended, as in RET (van der Sluis et al. 2007). Surprisingly, passive ultrasonic irrigation was used in only one of the included RET studies (McTigue et al. 2013). According to Widbiller et al. (2017), ultrasonic activation also enhances growth factor release from human dentine and that released bioactive proteins may act as autologous supplements for regenerative endodontic treatment (Widbiller et al. 2017). Therefore, a suggested irrigation regimen for the treatment of infected immature permanent teeth should include irrigation with large amounts of NaOCl in low concentrations followed by irrigation with EDTA (Galler et al. 2016). However, the results from the included studies could not find any correlation of the use of passive ultrasonic irrigation and success rates.

CHX Chlorhexidine gluconate (CHX) was used as supplemental irrigation. Ng et al. (2011) found that the additional use of 0.2% chlorhexidine solution for irrigation reduced the success of treatment significantly (Ng et al. 2011). A possible explanation could be the interaction product between NaOCl and CHX, a precipitate containing para-chloro-aniline, which is reported to be cytotoxic (Basrani et al. 2007). Nevertheless, the findings in this study could not identify any independent effect of irrigation with CHX on outcome in both groups, which showed equivalent success rates.

### Antibacterial root canal dressing

As mentioned above, both of the included studies in the RET group used modified triple antibiotic paste (TAP) as an inter-appointment disinfecting agent. This method, developed by Hoshino et al. (1996), combines Ciprofloxacin, Metronidazole, and Minocycline (Hoshino et al. 1996). Although the previously mentioned paste has the ability to combat bacteria specific to endodontic infections, it can cause severe tooth discolorations. Therefore, Minocycline has been replaced with Cefaclor and was launched as a modified triple antibiotic paste (mTAP) (Thibodeau and Trope 2007).

TAP and mTAP have several drawbacks, including the lack of standardised mixing protocols. Different amounts of the drugs are ground into powders and mixed with sterile water or macrogol ex tempore and applied in the root canal. This application causes uneven distribution and excessive concentration problems, resulting in high cytotoxicity (Ruparel et al. 2012). Recently, several authorities discouraged the use of one of the components in mTAP, Ciprofloxacin, due to its severe side effects (The Swedish Medical Products Agency, The European Medical Agency, The Food and Drug Administration Agency).

Therefore, the current recommendations include calcium hydroxide as a disinfecting agent in regenerative endodontic treatment (Endodontology 2006). Calcium hydroxide has good antibacterial properties and has been shown not to be cytotoxic to periapical tissues and SCAP: s (Sjogren et al. 1991). In accordance with existing literature, the disinfection protocols used in the both study groups effectively eliminated bacterial infections as healing of apical periodontitis was achieved (Sjogren et al. 1991, 1997; Sundqvist et al. 1998). Nevertheless, little is known about the post-treatment microbiologic status of the treated teeth, because none of the included studies reported bacterial samplings as a standardised control method of disinfection.

### Treatment provider

All the included studies in the RET and MTA treatment groups were performed at specialist clinics either by residents supervised by experienced endodontists and paediatric dentistry specialists or by the specialists themselves. Moreover, several studies reported that endodontic and paediatric specialists collaborated to provide non-compliant patients nitrous oxide/oxygen analgesia. All treatment procedures were performed with the aid of a surgical operating microscope. In this context, the treatment providers might have influenced the results and the external validity of the studies as operator experience is a key factor when it comes to outcome in endodontics (Alley et al. 2004). Root canal treatments consist of a series of independent steps, including isolation, access preparation, mechanical debridement, chemical irrigation, root canal dressing, and obturation. These factors have the ability to influence the treatment outcome (Ng et al. 2008). When the technical standard of root fillings is adequate, the healing rates are high (Sjogren et al. 1990; Bierenkrant et al. 2008).

### Outcome measures used in the included studies

It is important to analyse outcome measures as these are basis for evaluation of the treatment outcome of immature permanent teeth with pulp necrosis and apical periodontitis. Traditionally, endodontic outcome studies use strict criteria for success. They define successful treatment as absence of signs and symptoms of apical pathology, where the contours, width, and structure of the periodontal ligament around the treated tooth are radiographically normal (Sjogren et al. 1990, 1997; Ng et al. 2007). Factors associated with better tooth survival are the quality of the root filling, the length of root filling in relation to apex, optimal coronal restoration, and healthy preoperative apical status (Ng et al. 2010). It is generally accepted that optimal root filling lacks voids and ends within 0–2 mm from the radiographical apex (Frisk et al. 2008). In a meta-analysis, Ng et al. (2008) identified additional key factors that significantly improve the outcome of primary root canal treatment. These are preoperative absence of periapical radiolucency and satisfactory coronal restoration (Ng et al. 2008). However, we did not find a correlation between the preoperative presence of periapical radiolucency and the rate of success.

Often, radiographic assessment of treatment results is the only objective method used to evaluate endodontic treatment. Assessment is usually performed with intraoral radiographs using a variety of criteria to determine health of periapical. These strict radiographic criteria are established as a golden standard in the field of endodontic outcome measures (Sjogren et al. 1990, 1997). The most often used structured criterion for measurement of periapical status is the PAI index, which consists of five scores with cut-off points between PAI 2 and PAI 3 score for periapical health and disease (Orstavik et al. 1986). The analyses of the included studies showed that none of the RET studies reported PAI index at baseline or at follow-up while three of the MTA studies reported the use of PAI index both at baseline and at follow-up (Bucher et al. 2016; Mente et al. 2013; Kandemir Demirci et al. 2019). Therefore, in the vast majority of the studies, absence or presence of apical periodontitis at baseline and follow-up was identified as a radiographic outcome measure.

A more forgiving approach for outcome measure has been proposed by Friedman and Mor (Friedman, Mor 2004). Healing of periapical disease was compared to functionality or merely retention when endodontic treatment was weighed against tooth extraction and replacement with implant to provide relevant comparisons between the two treatment outcomes. This definition proposed the terms ‘functional retention’ and ‘survival’ as a specific goals of the individual patient and it includes teeth that may be healing or show signs of apical pathology. The third term, ‘success’, was adopted for teeth that have healthy apical status and are free of symptoms. Furthermore, the above-mentioned terms were applied for long-term evaluations and are less appropriate for short-term evaluations (< 10 years) (Dammaschke et al. 2003). The follow-up for the included RET studies varied between 24 and 49 months; however, large preoperative apical lesions could need a longer period to heal than small lesions (Sjogren et al. 1990). Therefore, in this study, the radiographic interpretation in terms of ‘success’ and resolution of periapical radiolucency should be interpreted with caution.

Therefore, we can say that the adopted terms of ‘functional retention’ and ‘survival’ may be seen as more loose criteria in the definition of endodontic outcomes. In other words, when we compare the outcomes of RET and MTA groups, we should bear in mind that the used outcome measures focus on different issues. In the RET group, the key outcome measure is the continuation of root development (root wall lengthening and root wall thickening); in the MTA group, the key outcome measure is the quality of the root filling. It could be justifiable for the included RET studies to use surrogate measures of treatment outcomes. Such measures are healing signs of local infection (sinus tract and abscess), healing of apical pathology, increase of root wall thickness, increase of root length, closure of apical, and absence of subjective symptoms. The study analyses showed that the majority of the cases were treated successfully and were retained throughout the follow-up. Furthermore, there were no statistical differences between the study groups.

Little is known about the impact of extruded MTA on the healing capacity of traumatised teeth with apical pathosis. Unfortunately, this topic has resulted in few studies with high scientific value; the results from several case reports and case series on extrusion of MTA have been inconclusive and dubious outcomes have been reported (Tezel et al. 2010; Tahan et al. 2010; Nosrat et al. 2012; Chang et al. 2013).

We found that three of the studies from the MTA group reported the level of root filling in relation to the root apex (Bucher et al. 2016; Demiriz and Hazar 2017; Mente et al. 2013). For example, one of the studies found that 30% of the root-filled teeth were underfilled or overfilled, but this did not affect the healing rate as 90% showed clinical and radiographical success (Bucher et al. 2016). Additionally, one study found no significant difference in periapical healing between overfilled and optimally filled teeth (Demiriz, Hazar Bodrumlu 2017). Unlike other studies, the results of this review showed that the level of root filling in relation to the root apex in the MTA study group did not affect the success rate considerably. This finding could be partially explained by the fact the MTA study group had good biocompatibility (Solanki et al. 2018) and partially by the described ability of MTA to stimulate deposition of mineralised tissue at the interface of the placement (Dreger et al. 2012).

In this review, the most consistent radiographic findings for both study groups were apical closure (apexification) and healing of periapical radiolucency (81–100%). The clinically meaningful findings include teeth being free from signs of local infection (sinus tract and abscess) and being free of symptoms. According to the adopted outcome measures, both RET and MTA groups showed high survival rates in the short term, ≤ 24 months (95%). A critical appraisal of these outcome measures would mean that the more forgiving measures were applicable for the RET study group (Friedman and Mor 2004).

### The importance of root maturation on outcome

Cvek (1992) described the problem of root fractures after prolonged intra-canal medication with calcium hydroxide in immature teeth. Root fracture has been recognised as one of the major complications in management of young necrotic immature teeth. The earlier the root development stage, the greater the risk of cervical root fractures (ranging from 77% in teeth with the lowest root development stage to 27% in the teeth with the highest root development stage). Cvek observed that the incidence of root fracture was associated with the root wall thickness rather than with prolonged intra-canal medication with calcium hydroxide. These findings were supported by several other studies (Kahler et al. 2018; Al-Jundi 2004). Further search in the literature did not identify studies reporting increased frequency of root fractures in immature teeth with open apices medicated with calcium hydroxide over varying periods (Morfis and Siskos 1991; Thater and Marechaux 1988).

It has been known that the application of MTA in the root canal facilitates an apical seal but does not promote continued root development. Similarly, none of the included MTA studies reported continued root formation and maturation. On the contrary, both the included RET studies reported accurate information on root wall widening and lengthening, significantly increasing root dimensions. The findings from the included studies showed that the reported overall incidence of root fractures was low (Table 10). Another finding is that an evaluation of the root maturation post-treatment was not possible to perform because not all of the included studies provided this information. In conclusion, based on the included information, it was difficult to establish a correlation between total medication time, continued root development, and the risk for root fractures.

### The effect of aetiology as a prognostic factor on outcome

Previous studies report that RET treatment of non-traumatic dental injuries, such as caries or developmental anomalies, results in a better prognosis than trauma cases (Lin et al. 2017; Cehreli et al. 2013). Severe trauma to the periodontal ligament induces inflammatory root resorption and may cause damage to the Hertwig epithelial root sheath as well as the apical papilla, compromising the outcome of regenerative treatment. Obviously, it is not possible to draw any conclusions on the effect of trauma on the outcome because the RET study group consisted of only two studies. However, both of these studies included both severe and non-severe trauma. No differences in overall success and survival rates were observed between severe and non-severe traumatic aetiology. In addition, no differences in the rate of root wall lengthening and thickening could be correlated to the nature of the trauma.

### Complications

The included studies indicated low frequency of occurrence of adverse effects. The most frequently reported drawback in both groups was tooth discoloration caused by MTA. Discoloration is an undesirable consequence of endodontic treatment and efforts should always be made to substitute agents that cause discolouring. Six out of seven of the included studies in both RET and MTA groups substituted grey MTA with white MTA. However, white MTA without bismuth oxide also causes discoloration in the presence of blood due to porosity of the cement or the iron absorption from haemoglobin (Torabinejad et al. 2018). Perhaps, MTA treatments are technique-sensitive procedures depending both on how experienced the treatment provider is and on the patient’s cooperation. Several difficulties—e.g. a child’s behaviour, proper visualisation, and the ideal placement of the material—are linked directly to the rate of discoloration.

### Strengths and limitations

A strength of this study is that it investigates a large number of immature permanent teeth with pulp necrosis and open apices (694 teeth) using both clinical and radiographic outcomes with a minimum follow-up of 24 months. In total, 116 teeth were treated in the included RET studies and 578 teeth were treated in the included MTA studies. In addition, the studies included in the MTA group also had longer follow-up and larger study sizes, resulting in better statistical significance.

The present review has, however, some limitations. The search was not expanded within unpublished literature (registries or theses). It was discussed that the risk for possible flaws in the design, conduct, analysis, and reporting of the results of unpublished studies would not have been detected unless submission to review process. Consequently, the authors agreed to include only published peer-reviewed studies.

Another limitation of this review is the fact that only seven studies matched the inclusion criteria of intervention studies on immature necrotic teeth with follow-up of at least 20 teeth for 24 months.

The number of included cases was unevenly distributed: compared to the RET group, the MTA group had approximately five times the total number of cases treated. In addition, the material that this review is based on consists of studies with different designs. Some studies had a retrospective design, which might limit the information if the treated teeth presented any signs or symptoms of pre-, intra-, or postoperative adverse effects. Another limitation is the fact that the periapical PAI index was not stated at baseline in all of the studies. The PAI index is considered to be accurate, reproducible, and suitable for observational studies (Orstavik et al. 1986). The index allows calibration between the observers and comparisons of the inter-observer validity (Huumonen 2002). In addition, the RET study group showed lack of standardised follow-up protocols despite the earlier suggested protocols (Jeeruphan et al. 2012; Jung et al. 2008; Wigler et al. 2013). These protocols have been adopted by the European Society of Endodontists. Feasible follow-up times are 3, 6, 12, 18, and 24 months and annually for five years (Galler et al. 2016). Unfortunately, few RET studies report follow-up periods longer than 24 months. To summarise, the heterogeneity of the study design of the included studies did not provide a definite answer for the outcomes of endodontic regenerative and apexification techniques.

In this review, clinical and radiographical outcomes of endodontic treatment in immature necrotic permanent teeth were compared with respect to regenerative and apexification techniques (calcium hydroxide and MTA apical plug). The knowledge gaps uncovered are based on the general absence of consensus within the research field. All the included studies claimed to have found one or more approaches for combating bacterial infection. Knowledge gaps were identified for regenerative endodontic treatment, apexification with MTA plug, and follow-up protocols. Further research is needed that examines treatment of traumatised immature permanent teeth with high-quality study design, larger study sizes, and longer follow-up periods. Future studies should predefine the root development stage and periapical index status (PAI index) at baseline and after treatment.

## Conclusion

In the present systematic review, the qualitative analysis revealed that both regenerative and apexification techniques had equal rates of success and survival and proved to be effective in the treatment of immature necrotic permanent teeth.

Endodontic regenerative techniques appear to be superior to apexification techniques in terms of stimulation of root maturation, i.e. root wall thickening and root lengthening.

Knowledge gaps were identified regarding the treatment and follow-up protocols for both techniques.

## Electronic supplementary material

Below is the link to the electronic supplementary material.

Supplementary file1 (DOCX 14 KB)

Supplementary file2 (DOCX 58 KB)
