# Supplementary material for: What is the best long-term treatment modality for immature permanent teeth with pulp necrosis and apical periodontitis?
Source: Eur Arch Paediatr Dent. 2021 Jan 8;22(3):311–40. doi: 10.1007/s40368-020-00575-1 (PMC8213569; doi:10.1007/s40368-020-00575-1)
Supplement: Supplementary file 1 — Supplementary file1 (DOCX 14 KB) [file 40368_2020_575_MOESM1_ESM.docx]

**Appendix 1: Search keys**

| Database |  |
| --- | --- |
| PubMed  n=1367 | ((((Regenerative Endodontics[MeSH] OR endodontic regenerat*OR regenerative endodontic* OR endodontic revital* OR endodontic revasculari* OR pulp revasculari* OR endodontic regeneration[MeSH:noexp] OR regenerat*OR revital*OR endodontic revitaliz*)) OR (open apex[Title/Abstract] OR open apices[Title/Abstract] OR traumatized immature teeth[Title/Abstract] OR non-vital immature teeth[Title/Abstract])) OR (``apexification*´´[Title/Abstract] OR MTA apical plug[Title/Abstract] OR calcium hydroxide apexification[Title/Abstract]) [NOT (In-vitro studies[Title/Abstract] OR animal studies[Title/Abstract] OR case report studies[Title/Abstract])](https://www-ncbi-nlm-nih-gov.proxy.kib.ki.se/pubmed?term=(((((Regenerative%20Endodontics%5bMeSH%5d%20OR%20endodontic%20regenerate%20*or%20regenerative%20endodontic*%20OR%20endodontic%20revital*%20OR%20endodontic%20revasculari*%20OR%20pulp%20revasculari*%20OR%20endodontic%20regeneration%5bMeSH:noexp%5d%20OR%20regenerate%20*or%20revival%20*or%20endodontic%20revitaliz*))%20OR%20(open%20apex%5bTitle/Abstract%5d%20OR%20open%20apices%5bTitle/Abstract%5d%20OR%20traumatized%20immature%20teeth%5bTitle/Abstract%5d%20OR%20non-vital%20immature%20teeth%5bTitle/Abstract%5d))%20OR%20(%60%60apexification*%5bTitle/Abstract%5d%20OR%20MTA%20apical%20plug%5bTitle/Abstract%5d%20OR%20calcium%20hydroxide%20apexification%5bTitle/Abstract%5d)))%20NOT%20(In-vitro%20studies%5bTitle/Abstract%5d%20OR%20animal%20studies%5bTitle/Abstract%5d%20OR%20case%20report%20studies%5bTitle/Abstract%5d)&cmd=correctspelling) |
| WoS  n=49 | (**``**Regenerative endodontic*´´ OR ``endodontic regenerat*´´ OR ``endodontic revital*´´ OR ``pulp revascularization´´)  *AND*  (``immature permanent teeth´´ OR ``open apex´´ OR ``root development´´ OR ``open apices´´) OR (``pulp necrosis´´ OR ``necrotic pulp´´ OR ``non-vital immature teeth´´)  AND  (``apexification*´´ OR ``calcium hydroxide apexification´´) OR ``MTA apical plug*´´ |
| Cochrane Library  n=169 | ``Regenerative Endodontics´´  AND  ``endodontic regenerat´´ OR ``regenerative endodontic´´ OR ``endodontic revital´´ OR ``endodontic revasculari´´ OR ``pulp revasculari´´  AND  ``open apex´´ OR ``root development´´ OR ``open apices´´ OR ``pulp necrosis´´ OR ``necrotic pulp´´ OR`` immature permanent teeth´´  AND  ``apexification´´ |
| Embase  N=222 | 'regenerative endodontic treatment' OR 'pulp revitalization' or 'endodontic regeneration' OR 'pulp revascularization' OR 'immature permanent teeth' OR ' open apex' OR 'root development' OR 'open apices' OR 'pulp necrosis' OR 'necrotic pulp' OR 'apexification' |
| Ovid (Medline)  n=387 | (regenerative endodontics or non-vital immature teeth or endodontic regeneration or apical revscularization).mp. [mp=title, abstract, original title, name of substance word, subject heading word, floating sub-heading word, keyword heading word, organism supplementary concept word, protocol supplementary concept word, rare disease supplementary concept word, unique identifier, synonyms  AND  (MTA apexification or calcium hydroxide apexification).mp. [mp=title, abstract, original title, name of substance word, subject heading word, floating sub-heading word, keyword heading word, organism supplementary concept word, protocol supplementary concept word, rare disease supplementary concept word, unique identifier, synonyms] |
