# Supplementary material for: What is the best long-term treatment modality for immature permanent teeth with pulp necrosis and apical periodontitis?
Source: Eur Arch Paediatr Dent. 2021 Jan 8;22(3):311–40. doi: 10.1007/s40368-020-00575-1 (PMC8213569; doi:10.1007/s40368-020-00575-1)
Supplement: Supplementary file 2 — Supplementary file2 (DOCX 58 KB) [file 40368_2020_575_MOESM2_ESM.docx]

**Appendix 2:**

The Risk Of Bias In Non-randomized Studies – of Interventions (ROBINS-I) assessment tool

(version for cohort-type studies)

**Version 19 September 2016**


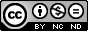


This work is licensed under a [Creative Commons Attribution-NonCommercial-NoDerivatives 4.0 International License](http://creativecommons.org/licenses/by-nc-nd/4.0/).

# ROBINS-I tool (Stage I): At protocol stage

## Specify the review question

| Participants | Children with immature necrotic permanent teeth |
| --- | --- |
| Experimental intervention | Regenerative endodontic treatment |
| Comparator | Apexification with CaOH2 or MTA apical plug technique |
| Outcomes | Apical closure; resolution of periapical lesion; increase in root length and thickness |

## List the confounding domains relevant to all or most studies

| Switching between compared interventions; Deviations from the treatment protocol; Non-adapted tool for radiographic measurements |
| --- |

## List co-interventions that could be different between intervention groups and that could impact on outcomes

| Deviations from the protocol and additional interventions if clinical symptoms were present |
| --- |

# ROBINS-I tool (Stage II): For each study

## Specify a target randomized trial specific to the study

| Design | Individually randomized / Cluster randomized / Matched (e.g. cross-over) |
| --- | --- |
| Participants | Children with immature necrotic permanent teeth |
| Experimental intervention | Regenerative endodontic treatment |
| Comparator | Apexification with CaOH2 or MTA apical plug technique |

## Is your aim for this study…?

| □x | to assess the effect of *assignment to* intervention |
| --- | --- |
| □ | to assess the effect of *starting and adhering to* intervention |

## Specify the outcome

Specify which outcome is being assessed for risk of bias (typically from among those earmarked for the Summary of Findings table). Specify whether this is a proposed benefit or harm of intervention.

| Apical closure; increase in root length and thickness |
| --- |

## Specify the numerical result being assessed

In case of multiple alternative analyses being presented, specify the numeric result (e.g. RR = 1.52 (95% CI 0.83 to 2.77) and/or a reference (e.g. to a table, figure or paragraph) that uniquely defines the result being assessed.

| Apical closure  Increase in root length  Increase in root thickness |
| --- |

## Preliminary consideration of confounders

Complete a row for each important confounding domain (i) listed in the review protocol; and (ii) relevant to the setting of this particular study, or which the study authors identified as potentially important.

#### “Important” confounding domains are those for which, in the context of this study, adjustment is expected to lead to a clinically important change in the estimated effect of the intervention. “Validity” refers to whether the confounding variable or variables fully measure the domain, while “reliability” refers to the precision of the measurement (more measurement error means less reliability).

| **(i) Confounding domains listed in the review protocol** | | | |
| --- | --- | --- | --- |
| **Confounding domain** | **Measured variable(s)** | **Is there evidence that controlling for this variable was unnecessary?*** | **Is the confounding domain measured validly and reliably by this variable (or these variables)?** |
| Switching between compared interventions | Protocol deviations | No information | No information |
| Severity of pre-treatment symptoms | Protocol deviations | No information | No information |
| Severity of post-treatment symptoms | Protocol deviations | No information | No information |
| Radiological failure | Root resorption | No information | No information |
| Clinical failure | Adjacent soft tissue pathology Pathologic mobility | No information | No information |
| Recurrence of trauma or caries | Radiological and clinical complications | No information | No information |
| Tool for radiographic measurements | Deviations in angulations in measurements of root length and thickness of dentinal walls | No information | No information |
| Selective reporting of outcome data | Measurements of failure and drop out rates | No | Yes |

* In the context of a particular study, variables can be demonstrated not to be confounders and so not included in the analysis: (a) if they are not predictive of the outcome; (b) if they are not predictive of intervention; or (c) because adjustment makes no or minimal difference to the estimated effect of the primary parameter. Note that “no statistically significant association” is not the same as “not predictive”.

Preliminary consideration of co-interventions

Complete a row for each important co-intervention (i) listed in the review protocol; and (ii) relevant to the setting of this particular study, or which the study authors identified as important.

#### “Important” co-interventions are those for which, in the context of this study, adjustment is expected to lead to a clinically important change in the estimated effect of the intervention.

| **(i) Co-interventions listed in the review protocol** | | |
| --- | --- | --- |
| Co-intervention | Is there evidence that controlling for this co-intervention was unnecessary (e.g. because it was not administered)? | Is presence of this co-intervention likely to favour outcomes in the experimental intervention or the comparator |
| Additional interventions if clinical or radiological symptoms present | No | Favour experimental |

## ROBINS-I Risk of bias assessment

**Response options for studies 1-5:**

**R1**-Chan et al (2017)

**R2**-Bucher et al (2016)

**R3**-Ree et al (2017)

**R4**-Demiriz et al (2017)

**R5**-Mente et al (2013)

Responses underlined in green are potential markers for low risk of bias, and responses in red are potential markers for a risk of bias.

|  | **Signalling questions** | **R1** | **R2** | **R3** | **R4** | **R5** |
| --- | --- | --- | --- | --- | --- | --- |
|  | | | | | | |
|  | 1.1 Is there potential for confounding of the effect of intervention in this study?   - 1. **If N/PN to 1.1:** the study can be considered to be at low risk of bias due to confounding and no further signalling questions need be considered | PN / N | PN / N | PN / N | Y / PY | PN / N |
|  | **If Y/PY to 1.1**: determine whether there is a need to assess time-varying confounding: | _ | _ | _ | Y / PY | _ |
|  | 1.2. Was the analysis based on splitting participants’ follow up time according to intervention received?  **If N/PN**, answer questions relating to baseline confounding (1.4 to 1.6)  **If Y/PY**, go to question 1.3. | _ | _ | _ | PN/N | _ |
|  | 1.3. Were intervention discontinuations or switches likely to be related to factors that are prognostic for the outcome?  **If N/PN**, answer questions relating to baseline confounding (1.4 to 1.6)  **If Y/PY**, answer questions relating to both baseline and time-varying confounding (1.7 and 1.8) | _ | _ | _ | _ | _ |

|  | **Questions relating to baseline confounding only** | **R1** | **R2** | **R3** | **R4** | **R5** |
| --- | --- | --- | --- | --- | --- | --- |
|  | 1.4. Did the authors use an appropriate analysis method that controlled for all the important confounding domains? | Y / PY | Y / PY | NI | N/PN | Y / PY |
|  | 1.5. Were confounding domains that were controlled for measured validly and reliably by the variables available in this study? | NI | Y / PY | NI | NI | Y / PY |
|  | 1.6. Did the authors control for any post-intervention variables that could have been affected by the intervention? | Y / PY | NI | N/PN | N/PN | Y / PY |
|  | **Questions relating to baseline and time-varying confounding** |  |  |  |  |  |
|  | 1.7. Did the authors use an appropriate analysis method that controlled for all the important confounding domains and for time-varying confounding? | Y / PY | N/PN | N/PN | N/PN | Y / PY |
|  | 1.8. **If Y/PY to 1.7**: Were confounding domains that were controlled for measured validly and reliably by the variables available in this study? | Y / PY | Y / PY | - | - | Y / PY |
|  | **Risk of bias judgement** | Moderate | Moderate | Serious | Critical | Low |
|  |  | | | | | |

| **Bias in selection of participants into the study** | | **R1** | **R2** | **R3** | **R4** | **R5** |
| --- | --- | --- | --- | --- | --- | --- |
|  | 2.1. Was selection of participants into the study (or into the analysis) based on participant characteristics observed after the start of intervention?  **If N/PN to 2.1:** go to 2.4  2.2. **If Y/PY to 2.1**: Were the post-intervention variables that influenced selection likely to be associated with intervention?  2.3 **If Y/PY to 2.2**: Were the post-intervention variables that influenced selection likely to be influenced by the outcome or a cause of the outcome? | PN / N | Y / PY | PN / N | Y / PY | PN / N |
|  |  | -  - | Y / PY  Y / PY | -  - | Y / PY  Y / PY | -  - |
|  | 2.4. Do start of follow-up and start of intervention coincide for most participants? | Y / PY | NI | Y / PY | NI | Y / PY |
|  | 2.5. Were adjustment techniques used that are likely to correct for the presence of selection biases? | NI | NI | Y / PY | PN / N | Y / PY |
|  | **Risk of bias judgement** | Low | Seroius | Low | Critical | Low |
|  |  |  | | | | |

| **Bias in classification of interventions** | | **R1** | **R2** | **R3** | **R4** | **R5** |
| --- | --- | --- | --- | --- | --- | --- |
|  | 3.1 Were intervention groups clearly defined?  3.2 Was the information used to define intervention groups recorded at the start of the intervention?  3.3 Could classification of intervention status have been affected by knowledge of the outcome or risk of the outcome?  **Risk of bias judgement** | Y / PY | Y / PY | Y / PY | PN / N | Y / PY |
|  |  | Y / PY | Y / PY | PN / N | PN / N | Y / PY |
|  |  | PN / N | Y / PY | PN / N | Y / PY | PN / N |
|  |  | Low | Moderate | Moderate | Critical | Low |
|  |  | | | | | |

| **Bias due to deviations from intended interventions** | | **R1** | **R2** | **R3** | **R4** | **R5** |
| --- | --- | --- | --- | --- | --- | --- |
|  | **If your aim for this study is to assess the effect of assignment to intervention, answer questions 4.1 and 4.2** | | | | | |
|  | 4.1. Were there deviations from the intended intervention beyond what would be expected in usual practice? | PN / N | PN / N | Y / PY | PN / N | PN / N |
|  | 4.2. **If Y/PY to 4.1**: Were these deviations from intended intervention unbalanced between groups *and* likely to have affected the outcome? | - | - | Y / PY | NI | - |
|  | **Risk of bias judgement** | Low | Low | Critical | Moderate | Low |
|  |  |  | | | | |

| **Bias due to missing data** | | **R1** | **R2** | **R3** | **R4** | **R5** |
| --- | --- | --- | --- | --- | --- | --- |
|  | 5.1 Were outcome data available for all, or nearly all, participants?  5.2 Were participants excluded due to missing data on intervention status?  5.3 Were participants excluded due to missing data on other variables needed for the analysis?  5.4 **If PN/N to 5.1, or Y/PY to 5.2 or 5.3**: Are the proportion of participants and reasons for missing data similar across interventions?  5.5 **If PN/N to 5.1, or Y/PY to 5.2 or 5.3**: Is there evidence that results were robust to the presence of missing data?  **Risk of bias judgement** | Y / PY | Y / PY | Y / PY | NI | Y / PY |
|  |  | PN / N | PN / N | NI | NI | PN / N |
|  |  | PN / N | Y / PY | Y / PY | PN / N | PN / N |
|  |  | - | Y / PY | NI | - | - |
|  |  | - | - | NI | - | - |
|  |  | Low | Moderate | Serious | Moderate | Low |
|  |  | | | | | |

| **Bias in measurement of outcomes** | | **R1** | **R2** | **R3** | **R4** | **R5** |
| --- | --- | --- | --- | --- | --- | --- |
|  | 6.1 Could the outcome measure have been influenced by knowledge of the intervention received? | PN / N | Y / PY | Y / PY | Y / PY | PN / N |
|  | 6.2 Were outcome assessors aware of the intervention received by study participants? | Y / PY | Y / PY | Y / PY | Y / PY | PN / N |
|  | 6.3 Were the methods of outcome assessment comparable across intervention groups? | Y / PY | Y / PY | Y / PY | Y / PY | Y / PY |
|  | 6.4 Were any systematic errors in measurement of the outcome related to intervention received? | PN / N | PN / N | PN / N | NI | PN / N |
|  | **Risk of bias judgement** | Moderate | Serious | Serious | Serious | Low |
|  |  |  |  |  |  |  |

| **Bias in selection of the reported result** | | **R1** | **R2** | **R3** | **R4** | **R5** |
| --- | --- | --- | --- | --- | --- | --- |
|  | Is the reported effect estimate likely to be selected, on the basis of the results, from... |  |  |  |  |  |
|  | 7.1. ... multiple outcome *measurements* within the outcome domain? | PN / N | PN / N | PN / N | Y / PY | PN / N |
|  | 7.2 ... multiple *analyses* of the intervention-outcome relationship? | PN / N | PN / N | PN / N | NI | PN / N |
|  | 7.3 ... different *subgroups*? | PN / N | PN / N | PN / N | Y / PY | PN / N |
|  | **Risk of bias judgement** | Low | Low | Low | Critical | Low |
|  |  |  |  |  |  |  |

| **Overall bias** | | **R1** | **R2** | **R3** | **R4** | **R5** |
| --- | --- | --- | --- | --- | --- | --- |
|  | **Risk of bias judgement** | Low | Serious | Serious | Critical | Low |
|  |  |  |  |  |  |  |


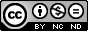


This work is licensed under a [Creative Commons Attribution-NonCommercial-NoDerivatives 4.0 International License](http://creativecommons.org/licenses/by-nc-nd/4.0/).
